# Neural Network-Based Mammography Analysis: Augmentation Techniques for Enhanced Cancer Diagnosis—A Review

**DOI:** 10.3390/bioengineering12030232

**Published:** 2025-02-24

**Authors:** Linda Blahová, Jozef Kostolný, Ivan Cimrák

**Affiliations:** Faculty of Management Science and Informatics, University of Žilina, 010 26 Žilina, Slovakia; jozef.kostolny@fri.uniza.sk (J.K.); ivan.cimrak@fri.uniza.sk (I.C.)

**Keywords:** mammography datasets, breast cancer detection, machine learning, data augmentation, BI-RADS classification, dataset imbalance

## Abstract

Application of machine learning techniques in breast cancer detection has significantly advanced due to the availability of annotated mammography datasets. This paper provides a review of mammography studies using key datasets such as CBIS-DDSM, VinDr-Mammo, and CSAW-CC, which play a critical role in training classification and detection models. The analysis of the studies produces a set of data augmentation techniques in mammography, and their impact and performance improvements in detecting abnormalities in breast tissue are studied. The study discusses the challenges of dataset imbalances and presents methods to address this issue, like synthetic data generation and GAN augmentation as potential solutions. The work underscores the importance of dataset design dedicated for experiments, detailed annotations, and the usage of machine learning models and architectures in improving breast cancer screening models, with a focus on BI-RADS classification. Future directions include refining augmentation methods, addressing class imbalance, and enhancing model interpretability through tools like Grad-CAM.

## 1. Introduction

Medical data is an essential component of medicine for research and healthcare advancement due to the vast amount of information it carries. With the increasing complexity and availability of medical data, understanding its origin, procedures, and annotations is essential for processing and analyzing it. This paper provides a background on medical data and its usage in neural network processing, specifically focused on oncological data. It covers the medical background on oncology, the cancer development process, and standard annotations for findings, diagnosis, treatment, screening methods, and description of chosen data type in oncology. Understanding the origin, procedures, and annotations of medical data of this type is essential for accurate data processing and analysis.

It is vital for medical data to encompass all relevant factors that need evaluation during diagnostics and future research. Such data must comply with established standards to ensure consistency, comparability, and reproducibility, which are important for building upon existing research. Given that medical data often contains sensitive personal information, prioritizing privacy and security is crucial when accessing or storing this data.

Moreover, ethical and legal considerations are paramount in the use of medical data. Medical research seeks to balance individual rights with societal benefits, as patients consent to share their medical information to enhance collective knowledge, ultimately leading to improved healthcare outcomes. To aid this balance, various frameworks and governance guidelines have been created.

Medical data plays a crucial role in healthcare, influencing patient safety, treatment quality, and chronic disease management. By analyzing historical findings and statistics, healthcare providers can develop less invasive surgical methods and enhance patient survival rates. Recently, this data has been pivotal in drug discovery and predicting epidemiological trends, especially during the pandemic.

A substantial amount of medical data is utilized in various research topics, including case-control studies and clinical trials. The shift towards personalized medicine reflects a growing focus on individual patient needs and the progression of their conditions over time. Additionally, advancements in technology have allowed for the exploration of telemedicine and artificial intelligence in healthcare, both of which are beginning to revolutionize the field. These innovations promise to create new opportunities, reduce costs, and improve the effectiveness of medical services and personalized treatment plans in the future.

## 2. Medical Background

Oncology is a medical field focused on the study and treatment of cancer and tumors, derived from the Greek word ’onkos’ meaning mass, and the Latin term for tumor [1,2]. It encompasses three main branches: radiation oncology, which employs radiation therapy; surgical oncology, which involves surgical interventions; and medical oncology, which utilizes drugs, hormones, or chemotherapy for treatment [3].

Cancer is characterized by the abnormal and uncontrolled growth of cells, with over 200 distinct types identified, making it a highly complex disease [4]. Annually, around 12 million individuals globally receive a cancer diagnosis, with estimates suggesting that one in five people will develop cancer in their lifetime. Mortality rates differ by gender, with approximately one in nine men and one in twelve women dying because of the disease [4]. Projections from the World Health Organization indicate a 77% increase in cancer cases by 2050, placing significant burden on healthcare systems [5].

In Europe, the Global Cancer Observatory reported over 4.8 million new cancer cases and more than 2.2 million deaths in 2022, with a total of about 14.7 million prevalent cases over five years. Age-standardized incidence rates reveal that men have a higher rate of 307.6 cases per 100,000, compared to 241.5 for women [6]. The cumulative risk of being diagnosed with cancer before age 75 is 30.8% for men and 23.7% for women. Mortality rates also reflect this imbalance, with men facing a 14.4% risk of dying from cancer before 75, compared to 8.9% for women [7].

Geographically, cancer incidence is notably higher in Northern and Western Europe, while Eastern European countries report elevated mortality rates. The most commonly diagnosed cancers include lung, breast, colorectal, prostate, stomach, and liver cancers, with lung cancer being the leading cause of death among men [8]. For women, breast cancer has the highest incidence and mortality rates, followed by lung and colorectal cancers [8]. Overall, the statistics signify the impact of cancer on public health and the need for advanced research and treatment advancements.

## 3. Mammography as a Cancer Screening Method

### 3.1. Screening in Oncology

Preventive measures and routine screenings are crucial in mitigating the harmful effects of cancer. These strategies not only aid individuals by facilitating early detection and reducing healthcare costs and risks, but also enhance public health by decreasing cancer incidence and mortality rates. Oncology employs various screening techniques tailored to specific cancer types. Traditional methods, like mammography, have been in use for years, though they often yield complex results. Conversely, ongoing research is focused on developing screening and prevention methods that are less invasive, more affordable, and more accommodating for patients.

### 3.2. Mammography

Mammography is a prevalent technique for breast cancer screening and diagnosis, utilizing X-ray technology to visualize breast tissue and detect lesions before they can be felt. Research indicates that regular mammography significantly lowers breast cancer incidence and mortality rates, with studies showing that it can identify cancer in approximately 9 out of every 1000 women [9].

Recent advancements in mammography include digital mammography, which produces digital images that can be easily shared and stored, and breast tomosynthesis, which creates three-dimensional images for better analysis and reduces the need for follow-up exams [10]. Additionally, computer-aided detection (CAD) systems enhance the identification of abnormalities by analyzing digital images for variations in tissue density and other indicators. The integration of artificial intelligence also holds promise for improving the accuracy of breast lesion assessments.

The primary advantage of mammography is its ability to detect carcinoma in situ, alongside its low radiation exposure, which minimizes side effects and ensures no residual radiation remains post-examination. However, there are risks associated with mammography, including radiation exposure, the potential for false positives or negatives due to breast density or human error, and the inapplicability of the procedure for pregnant women due to potential risks to the fetus [10].

## 4. Mammography Data

### 4.1. Available Datasets

Mammograms are X-ray images used for breast screening, typically involving two or more images per breast taken from craniocaudal (CC) and mediolateral oblique (MLO) angles. They help identify masses, calcifications, and other abnormalities, with calcifications categorized as either macrocalcifications, which are generally harmless, or microcalcifications, which can indicate early-stage cancer if clustered [11].

To assess mammogram quality, specific criteria must be fulfilled: all glandular tissue should be visible without skin folds or movement, and the images must include essential details like date, patient and radiographer identification, and side markers. The nipple should be centrally positioned in CC views, while MLO views must also capture the inframammary fold and pectoral shadow [11].

Breast density is categorized into four levels (a–d), where category ’a’ represents breasts primarily composed of fatty tissue, while category ’d’ signifies those with very high fibroglandular density [11].

**Figure 1:**
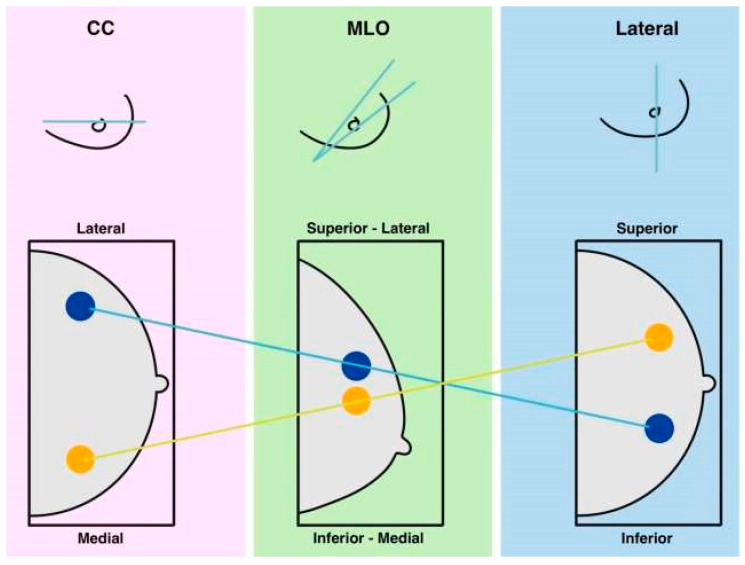
Lesion location on the CC, MLO, and ML views [11].

To be considered significant, masses in breast tissue must be visible in at least two imaging views, as shown in Figure 1. These masses can appear in various shapes and sizes, with clear boundaries visible on mammograms. Generally, masses that look abnormal have a higher likelihood of being dangerous. Calcifications scattered throughout the breast are typically benign. However, those that are concentrated in a specific region (diffused across the breast but not within the ducts) are usually non-invasive. In contrast, segmental (within ducts), linear (aligned within a duct), or clustered calcifications (more than five within 1–2 cm of tissue) are strong indicators of malignant (invasive) findings [12].

After analysis, mammograms are classified using the BI-RADS (Breast Imaging Reporting and Data System) evaluation system, which outlines various levels of findings. The different BI-RADS levels are described in Table 1.

Breast tumors that are cancerous often have an irregular shape. Unlike cysts, these tumors do not move and are usually painless. A BI-RADS Score of 6 is assigned only after a tumor is confirmed to be malignant [12].

Some of these factors can be important in mammogram evaluation. In breasts with higher tissue density (e.g., categories C and D), tumor visibility is lower, making lesion detection more difficult. On the other hand, breasts with higher density are more likely to develop breast cancer compared to categories A or B. Another issue is evaluating malignancy based on the BI-RADS score. BI-RADS categories 3 and 4, in particular, can sometimes be assessed as benign but turn out to be malignant, or vice versa. This is why having histopathology results as ground truth is crucial, as they clearly indicate whether a finding is benign or malignant.

There are multiple subtypes of breast cancer, depending on the location and severity. The four most common subtypes include Ductal Carcinoma In Situ (DCIS), Invasive Ductal Carcinoma (IDC), Invasive Lobular Carcinoma (ILC), and Triple-Negative Breast Cancer (TNBC). DCIS is an early-stage tumor that occurs in the milk ducts and has not yet become invasive, but can later develop into cancer. IDC is the most common type of breast cancer, spreading beyond the milk ducts and considered invasive. Another invasive subtype is ILC, which originates in the lobules and spreads to nearby structures. TNBC is an aggressive type of cancer that lacks hormone receptors and therefore requires a different treatment approach.

### 4.2. Selected Datasets and Their Annotations

There are multiple databases available, as presented in Table 2, either for the general public or upon request. These databases differ in many ways. Older databases generally provide less information about the findings and have lower-quality mammogram images, since they often contain traditional mammograms that were digitized. More recent databases include much more metadata, such as histopathology results, standardized details about stage evaluation, breast density, anomalies, or even episodes, which are sequences of a patient’s screenings at regular intervals. These sequences can be useful for analyzing how a patient’s lesion changes over time. Additionally, modern mammograms are higher quality and often include annotations in the form of binary masks around the lesion, which can be used to train machine learning models for breast cancer detection.

When choosing the appropriate databases, we considered several important points. The main goal was to use databases that are publicly available or can be obtained upon request and are well-annotated. We preferred datasets that provide BI-RADS scores for malignancy identification, histopathology labels, and lesion localization, either in the form of binary masks or bounding boxes. We also prioritized datasets with a large number of samples to support model generalizability and, optionally, those that include episodes. An overview of the selected databases is summarized in Table 3.

In our previous analysis, we decided not to use certain databases like INbreast [13] and MIAS [14] due to their lack of essential metadata, such as histopathology results, outdated format, and lack of standardization. Instead, we identified CBIS-DDSM [15] and OPTIMAM (OMI-DB) [16] as the most suitable databases for our research needs. The data from these databases has already undergone analysis and revision, with some modifications made. A comparative overview of these databases, made in a previous analysis of this topic, can be found in Chapter 2 of Ref. [17].

Moreover, we would now like to include new mammography databases, introduced recent studies, that may be relevant to our work. This includes the NYU Dataset [18], CSAW-CC [19], CMMD [20], and VinDr-Mammo [21]. However, we determined that the CMMD database is not appropriate for our study due to its lack of local annotations necessary for creating patches from full mammograms. Additionally, the NYU Dataset is private and inaccessible to us at this time, so it will not be explored further. Therefore, the rest of the work will focus on CBIS-DDSM, OPTIMAM, CSAW-CC, and VinDr-Mammo database.

We aim to use these databases, or, if possible, their combinations, to evaluate the impact of augmentation techniques on the performance of neural network models in binary and multiclass classification. We want to explore different classification tasks and address data imbalance. Additionally, we aim to identify a suitable data preprocessing sequence that could improve model performance by enhancing the quality of the input data. It is important to note that, in this process, we need to consider factors such as breast density distribution in the dataset and the types of lesions present (masses, calcifications, etc.).

The CSAW-CC dataset, a subset of the original CSAW dataset, was launched in 2020 and is based on a population study of women aged 40 to 74 who underwent mammography screening in Stockholm, Sweden, from 2008 to 2015. This dataset, designed for artificial intelligence research, includes a random selection of 873 positive and 7850 negative cases from an initial pool of 1103 positive and 10,000 negative cases. Each patient’s data encompasses all screening mammograms taken during the study period, along with metadata such as histological origin, tumor size, lymph node status, and cancer diagnosis. Notably, pixel-level annotations of lesions are available for identified tumors, while unobserved tumors are marked by a single point indicating their location [22].

VinDr-Mammo, released in 2022, is another significant dataset aimed at breast cancer detection, comprising 5000 screenings and 20,000 images collected from hospitals in Hanoi, Vietnam, between 2018 and 2020. The images are stored in DICOM format and include evaluations of breast density, lesion location, and BI-RADS assessments for malignant findings. Each sample has been reviewed by three radiologists, and the accompanying metadata provides details such as breast density, lesion category, BI-RADS findings, laterality, and bounding box annotations. The annotations categorize findings into various types, including masses, calcifications, asymmetries, architectural distortions, and other notable features like skin thickening and suspicious lymph nodes [21].

The application of machine learning in medical data heavily relies on annotations from medical experts for effective model evaluation and validation. Annotations in imaging can vary significantly; they may involve detailed delineation of the region of interest (ROI) or simpler geometric shapes like circles or squares. Precise annotations are particularly valuable for detection tasks, allowing for exact localization of findings, whereas simpler shapes are generally more suited for classification tasks where detailed positioning is less critical. Typically, precise annotations are represented as binary masks, where the white area indicates abnormal findings. Another precise annotation method involves listing coordinates that outline the region’s borders. In cases where the annotation is less exact, point coordinates are often utilized to define the borders. Common annotation techniques also include bounding boxes, defined by the top left corner’s coordinates along with the width and height, or for circular annotations, by specifying the center coordinates and radius.

#### 4.2.1. CBIS-DDSM

**Figure 2:**
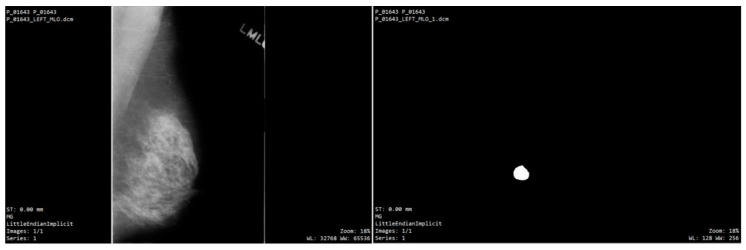
Sample from CBIS-DDSM database (left) and its respective annotation (right) [23].

The CBIS-DDSM database features comprehensive annotations ideal for various classification, detection, and segmentation tasks. Lesion annotations are provided as binary masks (Figure 2) that accurately outline the regions of interest (ROI) surrounding the findings, which are classified into masses or calcifications. These masks are stored in DICOM format, identical to the original mammograms. Furthermore, the dataset includes classification labels validated by histopathology, indicating whether the findings are benign or malignant. A key benefit of this database is the inclusion of the BI-RADS assessment score within the annotations [23].

#### 4.2.2. OMI-DB

The OMI-DB database is primarily designed for the classification and detection of cancer in mammograms, but it is not suitable for segmentation due to its use of surrounding rectangles to mark lesions. This method has the limitation of potentially including multiple lesion types within a single annotation. The findings are categorized into various types such as masses, calcifications, asymmetries, and architectural distortions, with classification labels derived from histopathology results indicating whether they are malignant or benign. However, the database lacks BI-RADS scores and breast density categories. A notable advantage of OMI-DB is its inclusion of regular screenings and patient episodes over time, which can provide valuable insights into the stages of cancer development [23].

#### 4.2.3. CSAW-CC

**Figure 3:**
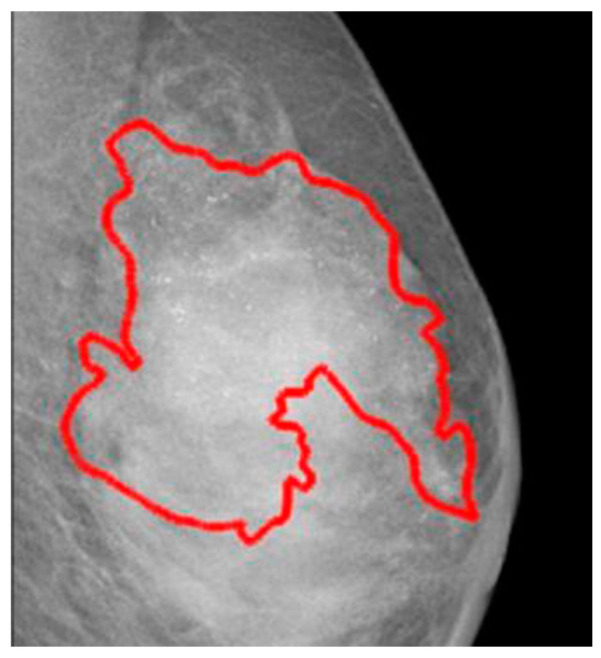
Example of ROI annotation (marked in red) in CSAW-CC database [19].

The CSAW-CC database offers various subsets suitable for cancer classification, detection, or segmentation in mammograms. However, not all samples feature detailed annotations with exact region of interest (ROI) contours, as presented in Figure 3. The pixel-level annotations encompass tumors identified at diagnosis, those noted during prior screenings, and areas where no tumors were present during earlier evaluations. These annotations provide measurements of tumor area (in square pixels) and the length of the major axis (in pixels) for both diagnosis and prior screenings. Tumors are categorized as invasive, in situ, or a combination of both. A limitation of the CSAW-CC database is the lack of specific BI-RADS scores. Similarly to the OMI-DB database, CSAW-CC’s primary advantage is its regular screenings, which might help to track tumor progression by using past annotations that show where tumors were located. Additionally, the database emphasizes real-world variability in age [19].

#### 4.2.4. VinDr-Mammo

**Figure 4:**
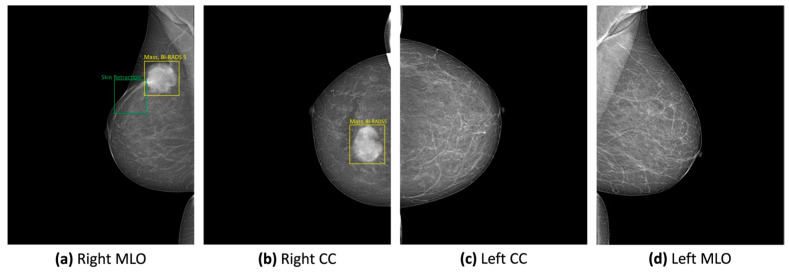
Example of bounding box annotations in VinDr-Mammo dataset [21].

The VinDr-Mammo dataset is designed for classifying and detecting breast cancer in mammograms, using rectangular coordinates to annotate lesions, as seen in Figure 4, which limits its use for image segmentation. It categorizes findings into mass, calcification, asymmetry, and distortion, incorporating BI-RADS evaluations and standardized breast tissue density assessments. However, a significant limitation is the lack of pathology-confirmed results, making the findings dependent on radiology experts. Additionally, the dataset primarily focuses on malignant cases, with only lesions classified as BI-RADS greater than 2 being annotated, leaving many benign findings unmarked. Despite these drawbacks, the dataset offers advantages such as public accessibility, accurate BI-RADS evaluations for density and classification, and a well-structured train/test data split that maintains a consistent sample distribution across various attributes, including patient age [21].

#### 4.2.5. Annotations in Other Previously Analyzed Databases

The study previously evaluated additional databases, specifically MIAS and INbreast. Among these, MIAS is deemed the least suitable due to its circular annotations around lesions, which limit its use to classification or detection tasks. It has several limitations, including outdated image formats, absence of BI-RADS scores, and lack of standardized breast density categories or histopathology results [23]. In contrast, the INbreast database features binary mask annotations for regions of interest (ROIs), which makes it applicable for segmentation, classification, and detection tasks. Both databases include various abnormalities such as masses, calcifications, asymmetries, and architectural distortions. A notable benefit of the INbreast database is the presence of BI-RADS score; it is, however, without histopathology findings [23].

## 5. Techniques for Data Processing and Analysis

Integrating machine learning models into mammography, and healthcare in general, requires a careful consideration of ethical concerns, especially in terms of data privacy and patient safety. Furthermore, when working with synthetic data, it is vital to address any biases that may occur. All of the available databases ensure that mammograms are properly de-identified and comply with regulations like GDPR and HIPAA. Removing sensitive metadata is an essential step to protect the patient. The data needs to be properly anonymized before any person utilizes it for preprocessing, training, or evaluating ML models.

### 5.1. Comparative Analysis of Traditional and Emerging Approaches in Mammography with the Use of Machine Learning Methods

Breast cancer detection in mammography has traditionally relied on radiologists’ expertise and experience, visually interpreting the mammograms and following standardized guidelines for diagnostics, such as the BI-RADS classification [12]. Traditional methods include X-ray mammography and breast ultrasound, which are commonly used for screening and diagnosis. Currently, more advanced techniques such as contrast-enhanced mammography and MRI are introduced as emerging tools. These provide an ability to improve early detection of cancer, especially for patients with high risk of cancer occurrence [10]. However, these approaches often depend on human interpretation, which can lead to subjective evaluation and diagnostic inconsistencies [9].

Machine learning-based methods offer a different approach by automating the process of feature extraction in the samples, and classification, potentially reducing variability in diagnosis. The models trained on large mammography datasets with high variability can detect complex patterns, or features connected with malignancy, which are not easily identified by a doctor’s visual observation [17,24].

The principal difference between traditional and machine learning-based approaches is that traditional methods rely on human and predefined rules for image analysis, while ML models, especially neural networks, identify complex relationships without rule-based training. On the other hand, such models then require careful validation and approval, to be used in clinical diagnostics. Overall, while traditional mammography remains the primary method for breast cancer detection, machine learning can contribute to automated decision-making, a reduction of diagnostic errors, and optimization of the workflow.

In addition to our focus on data augmentation, it is essential to delve into factors such as algorithm selection, feature engineering, and model evaluation to develop robust diagnostic tools for mammography. Convolutional neural networks (CNNs) have become the standard in image analysis because of their ability to learn features from raw data [15,21]. Recently, architectures such as transformer-based models have also been explored, since they appear to be better at handling complex, high-resolution images. To select the most appropriate algorithm, researchers need to analyze the characteristics of the dataset, define the specific clinical task they want to solve, and assess the computational requirements.

While neural network models excel at feature extraction and integration of features, feature engineering can still improve model performance. For instance, providing the model with additional data such as lesion morphology, calcification patterns and their distribution, breast density, or even findings from previous screenings have proven beneficial and has led to improved accuracy [23]. This approach allows models to benefit from both automated learning and the knowledge that comes from an expert in oncology.

Defining a reliable model evaluation system is also one of the most important aspects, as it determines whether the model can be used at all. Commonly used metrics such as accuracy, sensitivity, specificity, and AUC-ROC are essential for evaluating performance; however, in certain contexts, specific evaluation criteria may be set. For example, high sensitivity is required to ensure that malignant cases are not classified incorrectly, while high specificity is needed to minimize false positives and thereby reduce the number of unnecessary biopsies. Additionally, validation on independent and diverse datasets (i.e., from different databases) is crucial to confirm the model’s performance across various populations and imaging conditions. Moreover, the evaluation should involve cross-validation and analysis of failure cases to understand the limitations of the model, which can be beneficial for iterative refinement of both the input dataset and the trained model. Such an iterative process ensures that the results are not solely based on experimental performance but are carefully verified.

### 5.2. Principles of Dataset Design and Its Impact on Machine Learning Outcomes

When creating a dataset for machine learning, several key factors must be taken into account, applicable to both medical and technical fields. The principles for constructing an effective dataset are elaborated in detail in Ref. [11].

Firstly, the dataset’s relevance is paramount; it should directly relate to the research question and the specific problem being addressed, necessitating careful selection and analysis of the data. Moreover, the dataset must be diverse and representative, capturing the variability of the population relevant to the study, including factors such as gender, age, and ethnicity. It should also encompass a range of medical conditions to ensure all patient groups are included. Quality and accuracy are critical; the dataset must be free from errors, inconsistencies, or artifacts. This may require verification of annotations by experienced professionals to ensure precision. Completeness is another vital aspect; the dataset should contain all necessary information and labels for effective analysis and application of machine learning algorithms. In cases of small datasets or missing information, further investigation or mathematical approximations may be needed to address gaps. A uniform distribution of samples is essential for the effective functioning of machine learning algorithms, ensuring that the model has adequate representation from all classes within the dataset to accurately predict outcomes for new samples. Scalability is also important, allowing for the addition of more data in the future to support ongoing research or validation efforts. The dataset should be designed to be expandable, potentially facilitating longitudinal studies that track patient changes and disease progression over time. Lastly, comprehensive documentation and metadata are crucial for fostering collaboration and providing clarity regarding the dataset’s content and context. This includes detailed records of any modifications or removals of damaged samples, along with justifications for these actions.

The quality and characteristics of a dataset significantly influence the outcomes of machine learning algorithms, affecting both computational complexity and predictive performance. High-quality, well-annotated data enhances model accuracy and reliability, while a diverse dataset boosts generalizability to new data, which is essential for clinical applications. A thoughtfully designed dataset aids in error analysis and identifying model weaknesses, facilitating iterative enhancements. Additionally, clear annotations and thorough documentation enhance the interpretability of model results, allowing for informed suggestions for future improvements. Interpretability is especially critical in medical contexts to ensure acceptance in clinical settings.

### 5.3. Data Augmentation Techniques Overview

Data augmentation is a valuable method for expanding datasets used in neural network training, ultimately enhancing model accuracy. Various techniques are employed in imaging data augmentation, each with distinct impacts, as detailed in reference [24].

One fundamental technique is geometric transformation, which encompasses operations like translation, rotation, cropping, and flipping. This method is favored for its simplicity and quick implementation, though it does incur computational and memory costs during processing and training.

Another approach is noise injection, a pixel-level augmentation that involves adding noise by slightly altering pixel intensities. Other pixel-level methods include blurring and sharpening images, which help create a more robust dataset, although the optimal amount of noise remains uncertain.

Kernel filters represent another augmentation type, utilizing square masks to average surrounding pixel values and smooth images. Techniques like Gaussian blur, median, and Laplacian filters are employed to create sharpened and blurred images, beneficial for detection tasks. However, a downside is the potential loss of detail, as some images may become excessively blurred.

Random erasing is a technique where random quadrangles in an image are replaced with solid colors from the image itself. By varying the shapes, sizes, colors, and positions of these quadrangles, a single image can be reused multiple times to enhance the dataset. This method encourages the network to focus on the entire image, reducing overfitting risks, though it may lead to the loss of critical information.

More advanced augmentation methods include the use of generative adversarial networks (GANs) and neural style transfer (NST). GAN-based augmentation aims to create new images that closely resemble the originals, making it difficult for a discriminator model to distinguish between them. This approach involves two models: one that adds noise and another that classifies the images, with the classifier’s loss function guiding the noise generator. While it produces new, similar data, its exploration is still limited.

Neural style transfer is a creative augmentation technique that modifies the style of one image while preserving the content of another. By learning features from a ’style image’ and applying them to a ’content image’ through a convolutional neural network, NST enhances dataset diversity, improving classification model generalizability. However, challenges arise when the style and content images originate from vastly different datasets.

Recent studies, such as Ref. [25], propose additional augmentation techniques and algorithms. Ref. [26] reviews various studies and their employed augmentation methods, highlighting combinations, customizations, and innovations in augmentation strategies.

The implementation of augmentation techniques may vary and be specific to their intended usage and the author’s preferences. Simple augmentation techniques, including flipping (horizontal or vertical), rotations (e.g., 90° or 180°), cropping, and brightness or contrast adjustments, are often implemented using standard image processing libraries (for instance, scikit, Pillow, and OpenCV). With this approach, multiple variations of each mammogram are created. These basic augmentation methods have low computational cost and help increase the dataset size, allowing models to learn different features; however, they do not increase the variability of lesions in the dataset.

In contrast, advanced methods include neural style transfer (NST) or various types of GANs, such as the conditional infilling GAN (ciGAN). The aim of the ciGAN is to create new synthetic image patches by strategically adding lesions to images of healthy tissue as well as removing lesions from patches. This way, class imbalance can be addressed and dataset variability increased by simulating tumor appearances [27]. The GAN approach involves training a generator to produce images that resemble real mammograms in such a way that doctors cannot distinguish between real and synthetic images. While training the generator, the discriminator evaluates the authenticity, ensuring that the synthetic images reflect the distribution of the original data.

Validation of these augmentation techniques can be performed in multiple ways. The most basic method is comparing the performance of classification models trained on the original dataset with models trained on the augmented dataset using metrics such as accuracy, sensitivity, specificity, and AUC-ROC [28]. Another validation method involves cross-validation and testing the model on a different dataset than the one it was trained and evaluated on, which can confirm that the augmented images improve model generalizability without introducing misleading artifacts [24]. A third validation approach is to present the augmented dataset to a mammography specialist and ask them to distinguish between original and augmented images, thereby providing feedback on the realism of the synthetic images. Additionally, tools for neural network explainability can be used to define mathematical formulas that measure the performance of the model on both the original and augmented datasets.

### 5.4. Classification Tasks

Classification in machine learning is a supervised learning technique that involves training a model on a labeled dataset to predict the class of new, unlabeled data based on its input features. This method is primarily used for predicting discrete labels and is mathematically represented by conditional class probabilities, defined in Equation (1), where the output, denoted as *y*, can take values from a finite set of classes (1 to M). The probability of a specific class, given an input *x*, is denoted as *p*.

*p*(*y* = *m*|*x*) (1)

Various models can be employed for classification, including decision trees, logistic regression, mathematical formulas, classification rules, and neural networks. Before classification, it is crucial to identify relevant attributes that influence the class determination, which aids in simplifying the model by eliminating irrelevant data [29].

In the context of practical and research medicine, the input comprises examination results, which can be numeric, textual, or image-based. Attributes such as visual features in images or specific substance measurements are used to inform the diagnosis. This chapter elaborates on three primary types of classification, binary, multiclass, and multi-label, while also addressing the challenge of data imbalance in classification tasks.

#### 5.4.1. Binary Classification

Binary classification is a subset of multiclass classification where there are only two possible output classes, typically represented as [−1;1], [0;1], or [*false*;*true*]. In this context, the hypothesis function is often modeled using the sigmoid function in Equation (2), particularly in logistic regression [30].

(2) $$\sigma (x)=\frac{1}{1+e^{-x}}$$

To assess the performance of binary classification models, various evaluation metrics are employed, including accuracy (Equation (3)), precision (Equation (4)), recall (Equation (5)), F1 score (Equation (6)), confusion matrix, and ROC-AUC score. A practical application of binary classification can be seen in medical diagnostics, where the goal is to determine if a patient is healthy or diseased.

(3) $$A=\frac{TN+TP}{TN+FP+TP+FN}$$

(4) $$P=\frac{TP}{TP+FP}$$

(5) $$R=\frac{TP}{TP+FN}$$

(6) $$F1=2\times \frac{Precision*Recall}{Precision+Recall}$$

#### 5.4.2. Multiclass Classification

Multiclass classification refers to a classification method where samples are assigned to one of multiple classes, specifically when the number of classes, denoted as *M*, exceeds two. There are two primary approaches to multiclass classification: ’one vs. all’ and ’one vs. one’. The ’one vs. all’ method employs *M* binary classifiers to distinguish one class from all others, while the ’one vs. one’ method requires a number of classifiers N (Equation (7)) equal to the combination of classes, with each classifier focusing on a pair of classes. The softmax activation function and categorical cross-entropy loss are typically used to develop the model’s hypothesis [31].

(7) $$N\times \frac{\left(N-1\right)}{2}$$

Evaluation metrics for multiclass classification are similar to those used in binary classification, including accuracy, precision, recall, F1 score, and confusion matrix. In this context, the confusion matrix captures all potential classification outcomes, with true positive (*TP*) values positioned along the main diagonal.

#### 5.4.3. Multi-Label Classification

**Figure 5:**
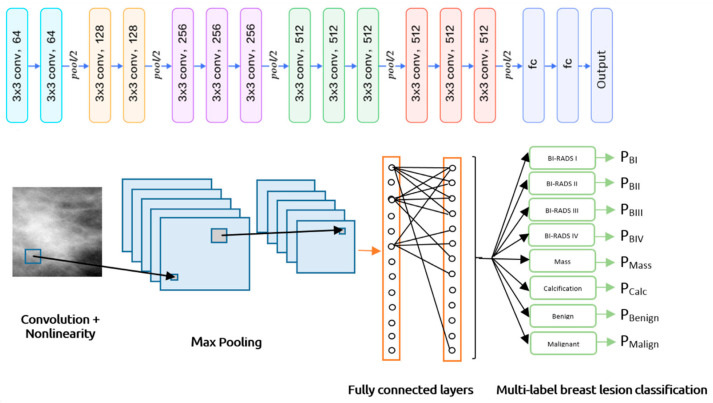
CNN architecture and stages of multi-label breast lesion classification [32].

Multi-label classification involves assigning multiple labels to data, as shown in Figure 5, distinguishing it from multiclass classification, where only one exclusive label is assigned. In multi-label scenarios, all relevant labels are applied, reflecting various characteristics and complex relationships. This classification approach typically employs binary cross-entropy and sigmoid activation to generate probabilities for each label.

For model evaluation, Hamming loss measures the number of incorrect predictions, while metrics like subset accuracy, balanced accuracy, precision, recall, and F1 score are often adjusted using micro, macro, or weighted averaging methods [33]. Multi-label classification finds applications in areas such as image annotation with multiple objects, categorizing text documents, and diagnosing conditions where multiple issues may coexist. An example of this can be seen in the CNN architecture for multi-label breast lesion classification.

#### 5.4.4. Imbalanced Classification

Imbalanced classification is a prevalent issue in machine learning, where the distribution of classes in datasets is often skewed. This imbalance can range from minor to severe, posing challenges for algorithms that typically assume equal representation across classes. Consequently, models struggle to accurately classify minority classes due to insufficient data to learn their patterns. Evaluating models in this context is crucial, as traditional metrics like accuracy can be misleading. Instead, metrics such as balanced accuracy, precision, recall, F1 score, and the ROC-AUC curve are more appropriate for assessing performance, particularly for minority class predictions [34].

In fields like medicine, imbalanced datasets are common, especially in diagnosing rare diseases or predicting unusual drug reactions. While the problem of class imbalance remains unresolved, several strategies can mitigate its effects. These include class weighting, random under-sampling, random over-sampling, synthetic over-sampling (like SMOTE), adjusting loss functions, and treating the problem as anomaly detection [34].

Class weighting adjusts the importance of classes during training, helping the model focus more on the minority class, though it can lead to overfitting with small datasets. Random under-sampling reduces the majority class to match the minority class size, but risks losing valuable information and diversity. Conversely, random over-sampling increases minority class samples through replication, avoiding information loss but potentially causing overfitting. Synthetic over-sampling generates new data points based on existing minority class samples, addressing the drawbacks of both under-sampling and over-sampling, though it can introduce noise if classes are not well-separated. Adjusting the loss function increases penalties for misclassifying minority class instances, encouraging better focus on these classes, though it requires careful tuning. Lastly, treating the minority class as anomalies allows for specialized outlier detection techniques to identify these rare instances effectively [35].

To optimize results in imbalanced datasets, a combination of these methods, often alongside data augmentation, should be explored.

### 5.5. Interpretation of the Impact of Dataset Adjustments

In the medical domain, understanding the decision-making process of machine learning models is as vital as assessing their performance. Model interpretation techniques are essential for elucidating how algorithms operate and ensuring their decisions are clinically relevant. Beyond mere accuracy, it is imperative to accurately identify critical medical areas such as tumors and other abnormalities in imaging.

Interpretation methods also facilitate the validation of dataset alterations, such as augmentation and resampling, allowing for the assessment of whether these changes enhance diagnostic performance. Techniques like Gradient-Weighted Class Activation Mapping (Grad-CAM), Layer-Wise Relevance Propagation (LRP), saliency maps, occlusion sensitivity, Shapley Additive Explanations (SHAP), and Local Interpretable Model-Agnostic Explanations (LIME) play a significant role in this process.

Grad-CAM generates visual explanations for CNNs by computing the gradients of the target class at the final convolutional layer. These gradients are averaged to assign importance weights to feature maps, which are then combined to produce a heatmap highlighting key regions influencing the model’s decision. This results in a class-discriminative localization map. Grad-CAM can be enhanced by integrating guided backpropagation for more detailed visualizations [36].

LRP interprets deep learning predictions by backpropagating relevance scores through the network, redistributing them across layers based on designed propagation rules. The method ensures that the total relevance score at each layer is preserved, aligning with a conservation principle. The output is a heatmap that highlights significant input features influencing the classification. LRP is applicable to various neural network architectures and is theoretically linked to Taylor decomposition [37].

Saliency maps identify the most influential pixels in an image for CNN classification. Unlike Grad-CAM, which focuses on regions, saliency maps operate at the pixel level, making them more detailed but often noisier. The process involves computing gradients of the class score with respect to the input image, followed by visualization as a heatmap. Due to their fine-grained nature, thresholding techniques can be applied to enhance interpretability [38].

Occlusion Sensitivity determines critical image regions by systematically masking parts of an image and observing the impact on classification scores. A colored square mask is moved across the image at different strides, recording changes in prediction scores. The compiled data forms a sensitivity map, where intensity represents the significance of each region for classification [39].

SHAP assigns an importance value to each feature in a classification model based on Shapley values from cooperative game theory. It evaluates the marginal contribution of a feature by comparing classification changes in subsets with and without it. The final contribution values are averaged to create a fair and additive distribution of importance across all features, ensuring their total sum matches the model’s prediction [40].

LIME explains machine learning predictions by locally approximating complex models with simpler interpretable ones. The method perturbs the input data, runs predictions, and assigns weights to the perturbed samples based on their similarity to the original data. A lightweight ML model is then trained on this modified dataset, and its feature weights are used to interpret the original model’s decision. LIME provides insights into feature contributions without relying on a specific model architecture [41].

These interpretation methods are crucial for aligning machine learning models with medical standards and enhancing their reliability in clinical applications.

### 5.6. Clinical Implications

Utilizing advanced data augmentation techniques in neural network models for mammography could truly help improve breast cancer detection. By making training data more diverse and realistic, these models can improve at spotting cancer earlier, which could lead to better outcomes for patients. Some studies have already shown that machine learning models can support radiologists in identifying malignant lesions, and artificial intelligence-assisted screening has helped improve early detection rates [42,43]. This suggests that implementing such models in routine practice could enhance diagnostic precision and efficiency.

Although, before these models can be used in real hospitals, there are a few big factors to deal with. One of the biggest aspects is regulation. All of the tools used in medicine, which applies to mammography as well, need to be approved by institutions like the Food and Drug Administration (FDA) and the Conformité Européene (CE) before they can be used in clinical practice.

Another significant point that we need to keep in mind is the bias in medical imaging datasets. It is a major concern because it can cause the ML models to perform differently across different demographic groups. For example, if a model is trained mostly on data from one gender, or one geographic location, its accuracy may drop when used for another. As an example, a study has shown that the neural network models can unintentionally learn demographic shortcuts, such as linking certain medical conditions to specific races or genders, which can lead to biased diagnoses [44]. While synthetic data, such as GAN-generated lesions, can address the imbalance in mammography datasets, it may not always capture the variability of real tumors. This drawback could affect diagnostic accuracy. Paying attention to these biases is essential in order to make sure the models are fair and reliable. This is also one of the reasons why we would like to use a diversity of datasets and their combinations in our study.

From a clinical perspective, integrating ML models into practice means they need to fit into the way doctors already work—helping rather than becoming a burden. These tools must connect smoothly with PACS systems so that doctors can use them without disrupting their workflow. The outcomes of the models also need to be explainable, not only because this is a fundamental requirement for all medical tools, but also because doctors need to understand why the model is making a certain decision. Furthermore, proper validation of the models is crucial to prevent false positives, which could lead to unnecessary biopsies.

Therefore, for neural networks and other complex models to be used daily in clinical mammography, these and many other challenges must be addressed. It is essential to tackle these challenges because models can only become a valuable tool for radiologists if they are trained and implemented correctly. In addition, these models should support, but not replace, radiologists, proving that doctor experience and knowledge is vital in decision-making.

## 6. Similar Studies

In order for machine learning algorithms to be effectively utilized in the medical field and other domains, they must exhibit a high degree of precision, reusability, reliability, and interpretability. Essentially, these algorithms need to replicate the capabilities of a human expert in their respective fields. Consequently, researchers are dedicated to training and optimizing these algorithms to enhance their performance. There exists a multitude of strategies that can be employed to refine the training process, which may lead to improved outcomes. However, it is important to note that enhancements are not guaranteed simply by applying these methods correctly. Researchers are tasked with the responsibility of thoroughly analyzing and evaluating various approaches and their effects, while also innovating new methodologies to ascertain which techniques yield the most beneficial results for their particular research objectives.

For the selection of studies, we set up a specific set of criteria to ensure relevance and consistency in our review. We included only studies published in the last five years to maintain up-to-date insights and follow the recent advancements in the field. Additionally, the selected studies had to use datasets from the databases defined in the begging of the study, to ensure either comparability with our previous work, or to provide new insights for us on the databases we would like to use in the future. Another requirement was that the study should involve a classification task and apply some form of augmentation technique. Ideally, we would like to have both of these requirements fulfilled, but for some studies, that offered a unique or larger contribution in a different aspect, and exceptions were made. Since our focus is on evaluating augmentation methods, we also paid attention to the variability of both the model architectures and augmentation techniques used. By selecting studies with diverse approaches, we aimed to capture a broader perspective on how different augmentation strategies impact classification performance on different classification tasks.

We are evaluating several aspects in the chosen studies. While it is difficult to find studies that meet all of the criteria, we also decided to include studies that may have a slightly different focus but are still significant from another perspective (e.g., a study focused on cancer detection instead of cancer classification, but providing valuable insights into augmentation). The first factor we consider is the mammography database used, as this determines the quality of the input given to the model. One of the most important aspects we evaluate is the augmentation used in the study. If the study applies multiple augmentation techniques, we focus on comparing them. If only one technique is used, we analyze its impact on the model’s performance compared to its previous performance on non-augmented data. Another key factor is the classification task, as binary, multiclass, and multi-label classification cannot be directly compared due to differences in complexity affecting model performance. Finally, we focus on the results obtained in the study, especially measured by standard metrics such as accuracy, sensitivity, specificity, or AUC-ROC, and the improvements in these metrics with the use of augmentation.

We decided to focus the review of similar studies on the augmentation techniques used, the classification task being solved, and the variety of available datasets and models. Most of the studies did not include technical details such as model hyperparameters (batch size, learning rate, weight decay, etc.), training time, or the computational resources used. Therefore, we could not include these details in our comparison. However, since this study focuses on data augmentation rather than model enhancement and tuning, we do not consider these missing details to be a critical drawback.

The study in Ref. [24] provides a comprehensive analysis of various augmentation techniques and their impact on mammogram classification performance. It explores basic methods like flipping and rotation, pixel-level modifications such as brightness and contrast adjustments, and advanced techniques including GAN-based augmentation and Neural Style Transfer. The research evaluates how these methods influence classification accuracy, sensitivity, and specificity in machine learning models, particularly convolutional neural networks, using datasets like MIAS, DDSM, and CBIS-DDSM. Findings indicate that simple geometric transformations can enhance classification accuracy by 5–10%, while advanced methods like GAN-based augmentation can lead to more substantial improvements, raising accuracy from approximately 80% to 94%. Sensitivity and specificity also saw notable gains, especially with GAN-augmented data, which boosted sensitivity by up to 15%. The study highlights the effectiveness of advanced techniques for small or imbalanced datasets and suggests that a combination of basic and advanced methods yields optimal results. It calls for further research to confirm these results across various datasets.

The research paper in Ref. [28] analyzes various image augmentation techniques and their effects on the performance of a deep learning model for classifying breast lesions, specifically calcifications and masses. The study aims to enhance classification accuracy and reduce overfitting by employing a transfer learning approach using the VGG-19 neural network, which has been pretrained on the ImageNet dataset. It also compares VGG-19 with VGG-16 and ResNet50. The research utilized the CBIS-DDSM dataset and implemented five basic augmentation techniques: rotation, horizontal and vertical flipping, zooming, contrast adjustment, and brightness adjustment. The methodology involved dataset splitting, image preprocessing and augmentation, training and fine-tuning the VGG-19 model, and evaluating its performance through accuracy, sensitivity, specificity, and AUC-ROC metrics. The findings revealed that augmentation significantly enhanced classification performance, achieving 90.4% accuracy, 94.01% sensitivity, 85.9% specificity, and 95.9% AUC-ROC. The findings indicate that the augmentation methods successfully minimized overfitting and enhanced the model’s capacity to generalize to unfamiliar data.

**Figure 6:**
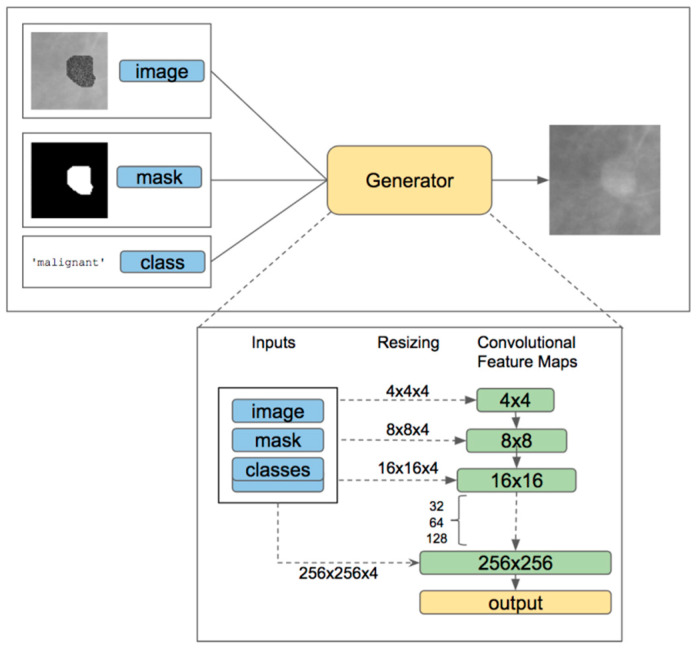
Architecture of the ciGAN network for synthetic image generation [27].

The study in Ref. [27] explored the use of Conditional Infilling Generative Adversarial Networks (ciGANs) to enhance mammogram classification by generating augmented image samples, as seen in Figure 6. This approach aims to tackle challenges like limited dataset sizes, class imbalances, and the high costs associated with expert annotations, utilizing the DDSM dataset. The researchers created synthetic patches from full mammograms by either adding lesions to non-malignant areas or removing them from malignant regions, focusing on 256 × 256 pixel patches that contain at least 75% breast tissue. The ciGAN model was trained using a combination of feature loss for image similarity, adversarial loss for realistic contours, and boundary loss for smooth transitions between original and generated areas. For classification, the ResNet-50 model was employed, trained on both the original dataset and a mix of traditionally augmented data (through methods like rotation, flipping, and rescaling) and ciGAN-generated data. The results indicated that ciGAN augmentation led to a 0.014 increase in AUC-ROC, enhancing classification performance compared to the original dataset. This suggests that ciGANs can produce realistic and contextually relevant images, aiding model generalization for small and imbalanced datasets.

The research in Ref. [45] examines how data augmentation influences the classification of breast masses in mammograms, utilizing the MIAS dataset that contains benign, malignant, and normal tissues. To combat data underfitting, the study employs basic geometric transformations like rotations and vertical flips. Images were cropped into 227 × 227 pixel patches centered on the mass and then normalized to 128x128 pixels. The performance of 24 machine learning models—including decision trees, support vector machines (SVMs), k-nearest neighbors (KNN), ensemble methods, and neural networks—was compared using both augmented and non-augmented datasets. The evaluation criteria included accuracy, sensitivity, specificity, precision, and F1 score for multiclass classification. Results showed an average accuracy increase of 8.26% with data augmentation, with neural network models particularly improving in detecting malignant masses (accuracy rising from 78% to 92%). The study concludes that even basic augmentation techniques can enhance the robustness and generalization of classification models in mammogram analysis. However, it notes limitations due to the focus on simple augmentation methods and the MIAS dataset.

The study in Ref. [46] introduced the application of Deep Convolutional Generative Adversarial Networks (DCGANs) to create synthetic breast mass images, aimed at enhancing mammogram datasets for better performance in computer-aided detection systems. Utilizing the OPTIMAM Mammography Image Database (OMI-DB), which contains a significant imbalance with 2215 mass patches and 22,000 normal tissue patches, the research involved cropping and resizing these patches to 128 × 128 pixels while normalizing them through histogram adjustments. The DCGAN’s generator maps a 200-dimensional latent vector to the original mass images, while the discriminator is trained using cross-entropy and non-saturated loss functions. Four data augmentation strategies were tested: using only original images, flipping original images, combining real images with DCGAN-generated ones, and merging both with additional flipping. A CNN was employed for classification, with the F1 score as the evaluation metric to assess breast mass identification accuracy. The strategy that combined DCGAN-generated images with flipping yielded the highest classification performance, increasing the F1 score by approximately 0.09 to nearly 99% compared to using only original images. This study demonstrates the effectiveness of DCGANs in generating realistic and diverse images, thereby enhancing classifier performance.

**Figure 7:**
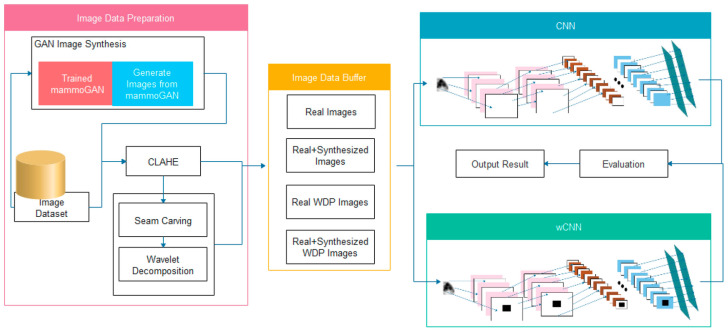
Diagram of the general approach used in Ref. [47].

The paper in Ref. [47] introduces a new convolutional neural network (CNN) architecture designed to enhance breast cancer detection through the integration of wavelet decomposition and transformation functions. The study aims to utilize Generative Adversarial Networks (GANs) for data augmentation, which helps improve feature extraction and tackle class imbalance. It merges two datasets, DDSM and MIAS, containing both benign and malignant samples. Various preprocessing techniques are employed, as presented in Figure 7, including Contrast Limited Adaptive Histogram Equalisation (CLAHE) for image contrast enhancement, seam carving to eliminate low-energy pixels while retaining essential features, and wavelet decomposition for extracting high-resolution, multi-level features. These preprocessing steps are vital for preparing images for the proposed wavelet-based CNN (wCNN) model, which innovatively substitutes the conventional ReLU activation function with wavelet transformations. Additionally, the study generates synthetic mammogram images using a GAN model, which are then combined with original images to create a more balanced training dataset. The enhanced wCNN model is evaluated on both the original and augmented datasets, with performance metrics including accuracy, sensitivity, specificity, precision, F1 score, and recall. The findings indicate that the wCNN architecture outperforms traditional CNNs, achieving around 99% accuracy, along with improvements in sensitivity and F1 score, reflecting better detection of true positives. However, the study also highlights a significant increase in computational complexity due to the intricate processes involved, such as combination of wavelet decomposition, seam carving, or GAN-based augmentation.

The article in Ref. [48] provides an in-depth examination of various data augmentation methods and their effects on the performance and generalization of a Convolutional Neural Network (CNN) tasked with differentiating between benign and malignant masses in mammography images. Utilizing the DDSM dataset and the AlexNet architecture, the study begins with a CNN pretrained on the ImageNet dataset, which is then fine-tuned using different augmentation techniques. These techniques range from basic geometric transformations, such as rotations, resizing, and flipping, to more sophisticated methods like filter bank responses and a texton-based approach. The texton-based augmentation involves creating a “texton dictionary” by filtering and clustering responses to capture texture patterns in regions of interest (ROIs). This dictionary enhances the CNN’s ability to identify subtle differences between benign and malignant tissues, resulting in improved classification accuracy. Specifically, the texton-based method achieved an accuracy of 87.04%, surpassing the 81% accuracy of simpler augmentation techniques. This indicates that texton-based models provide more detailed and discriminative features, allowing the CNN to differentiate between patient groups efficiently.

The paper in Ref. [49] analyzes various denoising techniques and their effects on deep learning models used for breast cancer detection, aiming to enhance accuracy, sensitivity, and specificity. Utilizing the DDSM dataset, the study first augments the data through simple rotations. It then implements three denoising methods: Deep Convolutional Neural Network (DnCNN), Wiener filter, and Median filter. For classification, a pre-trained CNN based on the AlexNet architecture is employed to distinguish between benign and malignant samples. The evaluation of classification accuracy shows an improvement from 88–89% for the original dataset to 90–94% for the denoised images. Sensitivity increased from 87–93% to 88–96%, while specificity rose from 83–91% to 84–96%. Notably, DnCNN outperformed the other methods in terms of denoising effectiveness. The findings underscore the significance of image pre-processing in enhancing model robustness and generalizability, particularly in medical applications. However, the study also notes the computational complexity associated with using DnCNN for denoising.

The paper in Ref. [50] discusses Attention-Guided Erasing (AGE), a new data augmentation method aimed at enhancing the accuracy and robustness of breast density classification in mammograms. Utilizing the VinDr-Mammo dataset, which consists of 20,000 digital mammography images categorized into four BI-RADS breast density levels, the study emphasizes classifying based on breast density rather than lesions. AGE employs attention maps from a Vision Transformer (ViT) model, pre-trained with the DINO self-supervised learning technique, to eliminate irrelevant background areas in images. This approach helps the model concentrate on dense breast tissue, crucial for precise classification. The classification task is executed using the DeiT-small ViT model, with AGE applied during training. The study evaluates AGE’s performance against traditional random erasing (RE) at different erasing probabilities and a non-augmented dataset, using the macro F1 score as the main metric. Results indicate that AGE significantly enhances classification performance, achieving a mean macro F1 score of 59% at an erasing probability of 0.6, compared to 55.94% without erasing and 56% with random erasing at a probability of 0.2. The findings suggest that AGE effectively directs the model’s attention to the most pertinent areas of the mammogram, although it does increase computational complexity and training duration.

**Figure 8:**
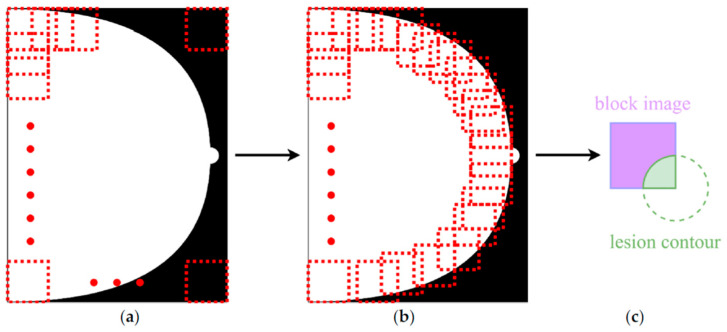
Patch generation process: (**a**) initial patch placement on the breast tissue only, (**b**) refined patch generation across the tissue, and (**c**) label assignment process used, as described in Ref. [51].

The study in Ref. [51] shifts focus from traditional dataset augmentation to the creation and assessment of a deep neural network (DNN) model designed to classify mammograms into specific BI-RADS categories, rather than simply distinguishing between malignant, benign, and healthy tissues. Utilizing the EfficientNet architecture and pretrained on the ImageNet dataset, the model was trained on a private dataset from Taiwan’s E-Da Hospital, which includes 5733 mammograms from 1490 patients, annotated with irregularly shaped points around lesions in JSON format. The mammogram images were segmented into 224 × 224 pixel patches, as shown on the Figure 8, to emphasize localized features relevant to each BI-RADS category. Patches were labeled based on two criteria: they must contain at least 90% breast tissue, and they must be categorized according to the cancer type present, following a hierarchical classification system from categories 5 to 1 (with Score 1 indicating a negative sample). The model demonstrated impressive performance metrics, achieving 94.22% accuracy, 95.31% sensitivity, 99.15% specificity, and a 97% AUC-ROC, particularly excelling in identifying higher-risk categories 4 and 5. However, the study notes challenges in accurately classifying BI-RADS category 1, indicating that further enhancements may be necessary to address this issue.

The study in Ref. [52] focuses on improving mass detection models for high-density breast tissue, which often leads to decreased performance due to the rarity of such cases in datasets. Researchers employed a CycleGAN model to create synthetic high-density images from lower-density ones, aiming to balance the dataset and enhance detection capabilities in dense breast mammograms. The study utilized multiple datasets, including CSAW-CC, OMI-DB, INbreast, and BCDR, categorizing mammograms according to BI-RADS breast density scores. The Deformable DETR model with ResNet-50 was used for feature extraction, and the inclusion of synthetic images in the training set resulted in improved sensitivity, precision, and detection accuracy for dense breast cases. The findings highlight the effectiveness of GAN-based augmentation in balancing datasets and enhancing the robustness of machine learning models. Notably, two radiology experts and an oncology surgeon evaluated the synthetic data and found it comparable to original samples, suggesting its potential for future clinical research.

The paper in Ref. [53] investigates the effects of advanced data augmentation techniques on imbalanced datasets in mammogram classification. It employs methods such as class weights, over-sampling, under-sampling, and Generative Adversarial Networks (GANs) to create synthetic lesions in mammograms. The augmentation process randomly generates three lesion types—masses, calcifications, and architectural distortions—using both benign and malignant samples from the original dataset. The research utilizes two recent datasets, CMMD and VinDr-Mammo, and assesses the performance of the ResNet-50 model on both in-distribution and out-of-distribution datasets to evaluate how class imbalance affects model performance. The evaluation metrics include accuracy, sensitivity, specificity, and AUC-ROC. Findings indicate a significant relationship between dataset class imbalance and model bias towards the majority class.

The evaluation of these studies is presented in Table 4.

The research in Ref. [24] highlights the significance of integrating both basic and advanced data augmentation techniques to enhance performance, particularly in small or imbalanced datasets. The study in Ref. [28] shows that data augmentation effectively boosts classification accuracy and mitigates overfitting. Another paper [27] discusses the use of conditional Generative Adversarial Networks (ciGANs) to create realistic synthetic data, which is beneficial for small datasets. The study in Ref. [45] demonstrates that even simple augmentation methods can greatly improve model performance in such contexts. The effectiveness of Deep Convolutional Generative Adversarial Networks (DCGANs) is also demonstrated in Ref. [46] in enhancing classifier performance for small datasets. Another study [47] reveals that combining wavelet transformations with GAN-based augmentation can lead to better classification accuracy. Advanced augmentation techniques, such as texton-based models, are acknowledged for their role in improving classification outcomes in Ref. [48]. Furthermore, a study on Deep Convolutional Neural Networks (DCNNs) [49] shows that denoising can significantly enhance classification accuracy, sensitivity, and specificity. The effectiveness of AGE in improving breast density classification is highlighted in Ref. [50], particularly in directing the model’s focus on relevant areas. Another study [51] indicates high classification accuracy in higher-risk BI-RADS categories, with room for improvement in lower-risk categories. The study in Ref. [52] employs CycleGANs to create high-density mammograms to enhance mass detection in dense breast tissue. The study in Ref. [53] examines how data augmentation affects imbalanced datasets in mammogram classification, highlighting its significance in clinical settings.

The key findings of the study are that basic augmentation techniques, such as geometric transformations, zooming, and brightness and contrast adjustments, may slightly increase classification accuracy. Advanced augmentation techniques, such as GANs for synthetic lesion generation, proved to be more efficient, mainly increasing sensitivity and overall accuracy by around 5 percent or more. Lastly, the algorithms introduced in recent studies are sophisticated and complex, achieving slightly better improvements than GANs, but often requiring high computational costs for efficient data processing.

In conclusion, GAN-based augmentation appears to be a good compromise between simple augmentation and more recent algorithms. However, its performance on small datasets may not improve the model as much as it would with a larger dataset. Another important takeaway from the analysis is that the outcomes of augmentation must reflect real-world conditions in terms of data distribution in order to be suitable for clinical use. Table 5 provides an overview of the conclusions from the analysis.

## 7. Discussion

This study demonstrates the effectiveness of advanced data augmentation techniques in improving machine learning models for breast cancer detection, especially in imbalanced datasets. The use of Generalized Annotated Neural Networks (GANs) combined with traditional methods has proven to be highly effective in improving sensitivity and specificity, crucial for early and accurate cancer detection. GAN-based augmentation significantly improved detection accuracy for dense breast cases, raising sensitivity by up to 15% compared to non-augmented datasets.

Synthetic data generation has also been found to mitigate class imbalance in medical imaging. GANs have allowed for the creation of realistic synthetic images that closely resemble original mammograms, enhancing model generalization. Combining basic geometric transformations with advanced methods like GAN-based augmentation led to substantial performance gains, particularly for small or imbalanced datasets.

The analysis showed that basic augmentation improved classification accuracy by 5–10%, while GAN-augmented data increased it by up to 20%. These results underscore the importance of combining different augmentation techniques for optimal results. Moreover, several studies have emphasized the role of synthetic data in addressing class imbalance, which is a common challenge in mammogram datasets.

However, while GAN-augmented data improves model sensitivity, the computational cost and complexity of training GANs must be acknowledged. The need for multiple rounds of data generation and model validation extends the training time and increases resource requirements.

The findings have important implications for the clinical deployment of machine learning models in breast cancer screening. The enhanced sensitivity and specificity offered by augmented models can improve early cancer detection rates. However, challenges such as misclassification of low-risk cases and increased computational burden must be addressed before these models can be widely adopted in clinical settings. Future research should focus on optimizing the balance between model complexity and clinical usability to ensure the benefits of advanced data augmentation translate into tangible improvements in patient outcomes.

There are, however, several limitations which must be considered. First, the dataset used for this study, though augmented, may not fully represent the diversity of breast cancer presentations in clinical practice. Second, the reliance on synthetic data generation introduces the risk of overfitting to augmented samples. Finally, further validation on more diverse and larger datasets is necessary to confirm the findings.

## 8. Future Directions

The conducted analysis and review of similar studies brought several opportunities for future research in the domain of mammography image analysis using machine learning models. To build on the findings of this research, future work should prioritize the areas of dataset design and augmentation, incorporation of background context, and focus on analyzing different classification tasks.

While this study already explored basic augmentation techniques, future work could benefit from further exploration into more sophisticated approaches to dataset augmentation. Enhancing the diversity of the generated mammogram images using augmentation may help address class imbalance issues and improve model performance. Experimenting with novel augmentation strategies to generate more diverse, high-quality training patches may further enhance the model’s generalization capabilities.

A limitation in the current patch-based approach is the exclusion of the broader image context. Future work should explore how including the surrounding tissue in the training process, rather than relying solely on isolated regions of interest, could improve model performance. By training models with both the lesion and background context, classifiers can develop a more nuanced understanding of mammographic images, potentially leading to improved detection accuracy, especially in ambiguous cases.

Although the current research explored the most common classification tasks in mammography, future studies could refine these approaches by investigating more granular classification strategies. Specifically, it would be useful to focus on BI-RADS categories with nuanced distinctions and evaluate how these contribute to more precise detection models. Additionally, balancing the dataset with augmentation outcomes could address the underrepresentation of certain BI-RADS categories in training data.

Additionally, we would like to explore and propose a specific method of model evaluation that could be expressed mathematically, based on the pixel-level annotations and Grad-CAM, or another interpretability technique with a defined threshold. As it will be an experimental method of evaluation, we will include details about this proposal in later studies.

By addressing these areas, future research can significantly enhance the performance and applicability of machine learning models in mammography, paving the way for more reliable and accurate breast cancer detection systems.

## 9. Conclusions

This study analyses and highlights the significant impact of advanced data augmentation techniques on improving machine learning models for breast cancer detection, specifically in mammography. By integrating conventional or advanced data augmentation methods, a considerable enhancement could be observed in model performance, particularly in addressing challenges related to class imbalance throughout all of the analyzed studies. Augmented datasets increased the accuracy, sensitivity, and specificity of the models, underscoring the importance of data augmentation to reduce bias and improve the reliability of AI-driven diagnostic tools in breast cancer screening.

While the results show promising advancements, this study also acknowledges the computational demands and complexity associated with advanced data augmentation techniques. The benefits of synthetic data generation, though evident in improving model generalization, come at the cost of extended training times and increased resource requirements. These factors must be considered when moving towards clinical applications of these models.

In conclusion, this research provides a foundation for the advancements and continued development of neural network-based mammography tools, offering improvements in cancer detection rates. However, further refinement and validation are necessary to ensure the clinical feasibility and robustness of these models in the real-world healthcare system.

## Figures and Tables

**Table 1:** BI-RADS score system for malignancy evaluation.

BI-RADS Mammography Evaluation Scale	Explanation
0	Inconclusive results; further imaging (MRI or ultrasound) is needed.
1	Negative; no abnormalities detected.
2	Benign findings such as calcifications or cysts.
3	Likely benign; follow-up screening is advised in six months.
4	Possible malignancy; a biopsy may be required.
5	Highly suspicious of cancer; biopsy is necessary.
6	Confirmed malignant tumor.

**Table 2:** Overview of available mammography databases and their issue year.

Available Database	Issue Year
MIAS	1994
DDSM	1997
BCDR	2012
INbreast	2012
CBIS-DDSM	2017
NYU Dataset	2019
CMMD	2021
OMI-DB (OPTIMAM)	2021
CSAW-CC	2022
VinDr-Mammo	2023

**Table 3:** Overview of selected databases and their attributes based on needs for this study.

Database	Annotations	Lesions	BI-RADS Score	Histopathology	Episodes
CBIS-DDSM	Binary masks	Masses, calcifications	Yes	Yes (benign/ malignant)	No
OMI-DB	Bounding box	Masses, calcifications, asymmetries, deformations	No	Yes (benign/ malignant)	Yes
CSAW-CC	Pixel-level	Masses, calcifications	No	Yes (malignant only)	Yes
VinDr-Mammo	Bounding box	Masses, calcifications, asymmetries, deformations	Yes	No	No

**Table 4:** Overview of the similar studies and their evaluation.

Study Title	Dataset	Models	Augmentation Techniques	Classification Type	Results
Image Augmentation Techniques for Mammogram Analysis	MIAS, DDSM, CBIS-DDSM	Various CNNs	Basic (flipping, rotation, brightness, contrast), Advanced (GAN-based, NST)	Binary, Multiclass	Basic methods improved accuracy by 5–10% GAN-based methods achieved 94% GAN-augmented data increased sensitivity by 15%
The Influence of Image Augmentation on Breast Lesion Classification Using Transfer Learning	CBIS-DDSM	VGG-19, ResNet50, VGG-16	Rotation, Flipping, Zooming, Contrast, Brightness	Binary	Accuracy: 90.4%, Sensitivity: 94.01%, Specificity: 85.9%, AUC: 95.9%
Conditional Infilling GANs for Data Augmentation in Mammogram Classification	DDSM	ResNet-50	GAN-based (ciGAN-generated patches)	Binary	AUC-ROC: 0.896 (with ciGAN), 0.014 increase from baseline
An Augmented Mammogram Image Dataset and Its Performance Analysis for Various Classification Models	MIAS	Decision Trees, SVMs, KNN, Ensemble Methods, Neural Networks	Rotation, Flipping	Binary, Multiclass	Accuracy improvement by 8.26% on average Significant improvement in neural networks for detecting malignant masses (up to 92%)
DCGANs for Realistic Breast Mass Augmentation in X-ray Mammography	OMI-DB	Fully Convolutional Network	GAN-based (DCGAN), Flipping	Binary	F1 score improvement by 0.09, reaching approximately 99% with combined GAN-generated and flipped images
A Novel Wavelet Decomposition and Transformation CNN with Data Augmentation for Breast Cancer Detection Using Digital Mammograms	DDSM, MIAS	Wavelet-based CNN (wCNN)	GAN-based	Binary	Accuracy: ~99% Significant improvements across all metrics
Investigating the Effect of Various Augmentations on the Input Data Fed to a CNN for Mammographic Mass Classification	DDSM	AlexNet	Geometric Transformations, Filter Bank Responses, Texton-based Models	Binary	Accuracy: 87.04% (Texton-based), 81% (Geometric), 85.8% (Filter Bank)
The Effect of Denoising on the Performance of DCNN for Mammogram Image Classification	DDSM	AlexNet	Denoising (DCNN, Wiener filter, Median filter)	Binary	Accuracy: 90.04–94.25%, Sensitivity: 88.04–96.52%, Specificity: 84.5–96.02% (with DCNN denoising)
Attention-Guided Erasing: A Novel Augmentation Method for Enhancing Downstream Breast Density Classification	VinDr-Mammo	DeiT-small ViT model	Attention-Guided Erasing (AGE)	Multiclass (BI-RADS breast density categories)	Macro F1 Score: 0.5910 (AGE), 0.5594 (no erasing), 0.5691 (random erasing)
A High-Performance DNN Model for BI-RADS Classification on Screening Mammograms	Private dataset from E-Da Hospital	EfficientNet	No Augmentation	Multiclass (BI-RADS cancer severity categories)	Accuracy: 94.22%, Sensitivity: 95.31%, Specificity: 99.15%, AUC-ROC: 97%
High-Resolution Synthesis of High-Density Mammograms: Application to Improved Fairness in Deep Learning-Based Mass Detection	CSAW-CC, OMI-DB, INbreast, BCDR	Deformable DETR and ResNet-50 for feature extraction	CycleGAN	Binary	Improved sensitivity by 12% and precision by 10% in dense breast tissue mass detection
Improving Mammogram Classification with GAN-Based Augmentation Techniques for Imbalanced Datasets	VinDr-Mammo, CMMD	ResNet-50	Class weights, under-sampling, over-sampling, GANs	Binary	Accuracy improved by 5%, sensitivity by 7%, specificity by 6%, AUC-ROC by 0.02

**Table 5:** Impact, usability, advantages, and disadvantages of the different levels of augmentation.

Augmentation	Accuracy Improvement	Dataset Size	Classification Type	Advantages	Disadvantages
Basic	Moderate (3–5%)	Smaller (up to 10,000 samples)	Binary, Multiclass	Simple and quick implementation	Little improvement
Advanced	High (above 5%)	Larger (above 10,000 samples)	Binary	High improvement	Hardware requirements, time
New Algorithms Introduced	High (above 5%)	Larger (above 10,000 samples)	Multiclass, Multi-label	Effective processing of vast datasets	Complexity, hardware requirements, limited verification options

## Data Availability

No new data were created or analyzed in this study.
